# Clofazimine, but Not Isoniazid or Rifampicin, Augments Platelet Activation *in vitro*

**DOI:** 10.3389/fphar.2018.01335

**Published:** 2018-11-20

**Authors:** Ronald Anderson, Annette J. Theron, Jan G. Nel, Chrisna Durandt, Moloko C. Cholo, Charles Feldman, Gregory R. Tintinger

**Affiliations:** ^1^Department of Immunology, Faculty of Health Sciences, University of Pretoria, Pretoria, South Africa; ^2^Institute for Cellular and Molecular Medicine, Faculty of Health Sciences, University of Pretoria, Pretoria, South Africa; ^3^Department of Haematology, Faculty of Health Sciences, University of Pretoria, Pretoria, South Africa; ^4^Tshwane Academic Division, National Health Laboratory Service, Pretoria, South Africa; ^5^Department of Internal Medicine, Faculty of Health Sciences, University of the Witwatersrand, Johannesburg, South Africa; ^6^Department of Internal Medicine, Faculty of Health Sciences, Steve Biko Academic Hospital, University of Pretoria, Pretoria, South Africa

**Keywords:** adenosine 5′-diphosphate, clofazimine, isoniazid, neutrophils, platelets, P-selectin, rifampicin, thrombin

## Abstract

Although the inclusion of the cationic amphiphilic, anti-mycobacterial agent, clofazimine, in the chemotherapeutic regimens of patients with multidrug-resistant tuberculosis (TB) has contributed to improved outcomes, concerns remain about the cardiotoxic potential of this agent. Accordingly, the current study was undertaken with the primary objective of investigating the effects of clofazimine, on the reactivity of human platelets *in vitro*, a seemingly unexplored, mechanism of cardiotoxicity. Platelet-rich plasma (PRP) prepared from the blood of healthy, adult humans was treated with clofazimine (0.625–10 mg/L), or the primary anti-TB agents, isoniazid and rifampicin (at final concentrations of 5 and 10 mg/L), followed by addition of either adenosine 5′-diphosphate (ADP) or thrombin and measurement of platelet activation according to the magnitude of expression of CD62P (P-selectin), as well as the CD62P-mediated formation of heterotypic neutrophil:platelet (NP) aggregates, using flow cytometry. Clofazimine, but neither isoniazid nor rifampicin, caused dose-related potentiation of both ADP- and thrombin-activated expression of CD62P by platelets, achieving statistical significance at threshold concentrations of 0.625 and 2.5 mg/L, respectively, as well as significant formation of N:P aggregates. These stimulatory effects of clofazimine on platelet activation were partly attenuated by pre-treatment of PRP with the membrane-stabilizing agent, α-tocopherol, possibly consistent with a membrane-disruptive mechanism. In conclusion, clofazimine, at concentrations within the therapeutic range, augments platelet activation *in vitro*, probably by a mechanism linked to membrane destabilization. If operative *in vivo*, these pro-thrombotic activities of clofazimine may predispose for development of microvascular occlusion, exacerbating an already existing high risk for development of TB-associated cardiovascular disease.

## Introduction

Recent developments in the antimicrobial chemotherapy of multidrug-resistant and extensively drug-resistant (MDR/XDR)-tuberculosis (TB) include recognition of the therapeutic efficacy of the “repurposed” riminophenazine agent, clofazimine (Aung et al., 2014; Tang et al., 2015; Trébucq et al., 2018) which appears to target both replicating and non-replicating *Mycobacterium tuberculosis* bacilli (Cholo et al., 2017). Concerns remain, however, about the risk for development of cardiac dysfunction during chemotherapy with this agent, specifically prolongation of the cardiac QT interval, an indicator of development of ventricular tachyarrhythmias and possibly cardiac arrest (Choudhri et al., 1995; Li et al., 2016; Wallis, 2016). The risk of drug-related cardiotoxicity in patients with TB may be further exacerbated by the fact that the infection *per se* is associated with systemic inflammation and a pro-thrombotic state, characterized by an increased risk of acute coronary events (Chung et al., 2014; Huaman et al., 2017). This risk may be intensified firstly by prolonged disease and extended chemotherapy, particularly in those patients with comorbid disease (Pepper et al., 2010) and, secondly, by the fact that clofazimine is often used in combination with other anti-mycobacterial agents known to possess cardiotoxic potential, specifically bedaquiline and delaminid (Tadolini et al., 2016; Wallis, 2016).

Importantly, however, little is known about the effects of clofazimine on the reactivity of human platelets, cells which are not only key mediators of thrombosis, but which are also major drivers of neutrophil-mediated inflammation, largely mediated via upregulated expression of the adhesion molecule, CD62P (P-selectin), stored in cytoplasmic α-granules (Wagner, 2005). In addition to pro-thrombotic activity, platelets also possess pro-arrhythmogenic potential (de Jong and Dekker, 2010). Accordingly, the current study has been undertaken with the primary objective of investigating the effects of clofazimine, as well as those of the first-line anti-TB drugs, isoniazid and rifampicin, on expression of the key mediator, CD62P, by agonist-activated platelets *in vitro*, as well as the modulatory potential of the membrane-stabilizing agent, α-tocopherol.

## Materials and Methods

### Ethics Committee Approval

Permission to undertake this study and draw blood from healthy, adult human volunteers (mean age 32.2 years; 26 females: 16 males) was granted by the Research Ethics Committee of the Faculty of Health Sciences, University of Pretoria in full compliance with the World Medical Association Declaration of Helsinki 2013 (Approval No. 116/2017). Prior written informed consent was obtained from all blood donors each of whom underwent a health check (including measurement of blood pressure) by an experienced, qualified nursing sister prior to the blood draw.

### Antibiotics and Other Pharmacologic Agents

Clofazimine, isoniazid, rifampicin, and α-tocopherol (vitamin E), were purchased from the Sigma Chemical Co. (St Louis, MO, United States). All of these agents were dissolved to stock concentrations in dimethylsulphoxide (DMSO), with appropriate solvent controls included in the assays of platelet activation described below.

Unless indicated, all other chemicals were also purchased from the Sigma Chemical Co.

### Platelet-Rich Plasma (PRP) and Buffy Coat Preparation

To prepare PRP, blood (anti-coagulated with five units of preservative-free heparin/mL blood) was centrifuged at 250 ×*g* for 10 min at room temperature within 15 min of venepuncture as described previously (Nel et al., 2016). The essentially erythrocyte- and leukocyte-free upper layer of PRP was decanted and used in the experiments described below. Buffy coat suspensions prepared by sedimentation of heparinised blood at 37°C were used for analysis of neutrophil;platelet (NP) heterotypic aggregate formation (Nel et al., 2017).

### Effects of the Test Agents on Platelet Activation

Platelet-rich plasma (20 μL) was added to 980 μL of Hanks’ balanced solution (HBSS, indicator-free, 1.25 mM calcium, pH 7.4) and incubated for 5 min at 37°C followed by addition of 1–2 μL clofazimine (0.625 – 10 mg/L, final), isoniazid or rifampicin (5 and 10 mg/L, final), or DMSO to control systems (0.1–0.2%, final), and the test tubes incubated for a further period of 10 min at 37°C. Thereafter, the potent, primary activator of platelets, thrombin (from human plasma, used at a final sub-maximal concentration of 0.31 NIH units/mL), or the secondary, autocrine activator, adenosine 5′-diphosphate (ADP, 100 μM, final maximal concentration), agonists of the platelet proteinase-activated receptors (PARs) 1 and 4 and the purinergic P2Y1 and P2Y12 receptors, respectively, or an equal volume of HBSS (background to detect spontaneous platelet activation), were added to the tubes. These were then incubated for a further period of 5 min at 37°C and processed immediately thereafter for analysis by flow cytometry. In an additional series of experiments limited to clofazimine-treated systems, the effects of pre-treatment of PRP with α-tocopherol (30 mg/L, final) on clofazimine (0.625 and 1.25 mg/L)-mediated potentiation of ADP-induced platelet activation were investigated.

### Measurement of Platelet Activation

Platelet activation was measured by flow cytometry as the proportion (%) of CD42a^+^ platelets expressing the α-granule-derived adhesion molecule, CD62P, considered to be a key mediator of platelet activation and pro-thrombotic activity (Wagner, 2005). Following incubation with the various antimicrobial agents, the platelet suspensions (PRP) were stained with 5 μl each of a murine anti-human platelet CD42a-phycoerythrin (PE)-labeled monoclonal antibody and an anti-human CD62P-fluorescein isothiocyanate (FITC)-labeled monoclonal antibody (both from Becton Dickenson, San Jose, CA, United States) to detect the total and activated platelet populations, respectively. After 15 min of incubation in the dark, the samples were analyzed on a Gallios flow cytometer (Beckman Coulter, Miami, FL, United States) and the results expressed as the percentage of activated platelets with 50,000 cells interrogated during each measurement. The gating and analytical strategies used in a typical experiment are shown in the Supplementary Figure S1.

### Neutrophil:Platelet (NP) Aggregate Formation

In this more limited series of experiments, the effects of clofazimine only at fixed concentrations of 2,5 and 5 mg/L on NP interactions were investigated. Buffy coat (20 μL), suspended in 980 μL HBSS, was processed as above for PRP and NP aggregate formation measured following activation with thrombin (0.31 NIH units/mL) or ADP (100 μM) as described previously (Nel et al., 2017). Following incubation, the cells were stained for 15 min at room temperature in the dark with a cocktail consisting of 5 μL of each of the following, murine, anti-human, fluorochrome-labeled monoclonal antibodies: CD16-allophyocyanin (Biolegend, San Diego, CA, United States), CD42a-PE (Becton Dickenson) and CD45-Krome Orange (Beckman Coulter) to enable detection of neutrophils, platelets and total leukocytes, respectively. This was followed by analysis of the cell suspensions at a slow flow rate using the Gallios flow cytometer. NP heterotypic aggregate formation was determined as CD16^+^/CD45^+^ neutrophils co-expressing CD42a. Results are expressed as the relative median fluorescence intensity (MFI) of CD42a as emitted by CD16^+^/CD45^+^ neutrophils as an index of the magnitude of the interaction of platelets with individual neutrophils.

### Expression and Statistical Analysis of Results

The results of each series of experiments are expressed as the mean values ± standard deviations (SDs) with the numbers of different donors and replicate experiments clearly indicated. Statistical analysis was performed using WinStat statistical software with levels of statistical significance calculated using the Mann-Whitney *U*-test for comparison of non-parametric data. A *P*-value of ≤0.05 was considered significant.

## Results

### Platelet and Leukocyte Counts

The mean numbers of platelets and total leukocytes in the PRP preparations were 262 ± 85 x 10^9^/L and 0.15 ± 0.1 × 10^9^/L, respectively, while the numbers of platelets and neutrophils in the buffy coat suspensions were 468 ± 219 × 10^9^/L and 4.6 ± 2.2 × 10^9^/L, respectively, (mean values ± SDs).

### Effects of the Test Antimicrobial Agents on Spontaneous and ADP- or Thrombin-Activated Expression of Platelet CD62P

**FIGURE 1:**
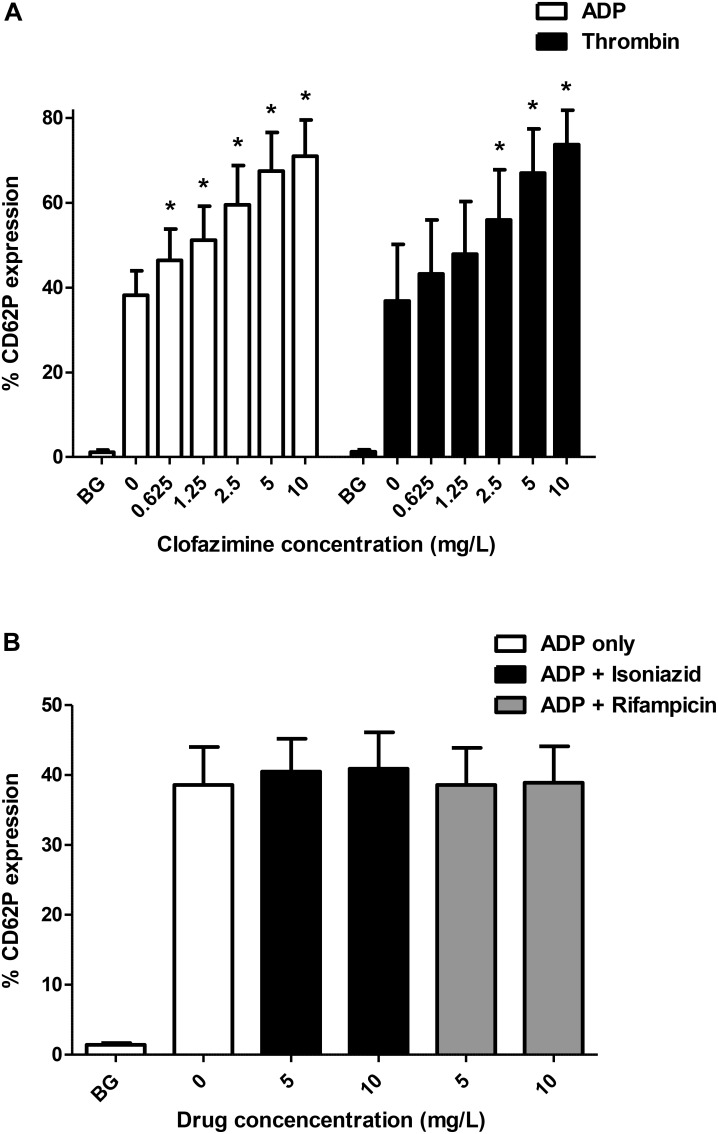
**(A)** Effects of clofazimine (0.625–10 mg/L) on CD62P expression on unstimulated (BG, background) platelets and cells activated with ADP (100 μM; data from 11 experiments using PRP from seven different donors) or thrombin (0.31 NIH units/mL; data from eight experiments using PRP from five different donors). The results are expressed as the % activated platelets. The effects of isoniazid and rifampicin at concentrations of 5 and 10 mg/L on ADP (100 μM)-activated expression of CD62P on platelets are shown in Panel **(B)** (data from five separate experiments). ^∗^*P* < 0.05 for comparison of clofazimine-treated ADP- or thrombin-activated systems with the corresponding clofazimine-free control systems.

Of the 3 agents tested, only clofazimine was found to cause a statistically significant augmentation of both ADP- and thrombin-activated expression of CD62P by platelets as shown in Figure 1A. Clofazimine-mediated augmentation of CD62P expression attained statistical significance at threshold concentrations of 0.625 and 2.5 mg/L for ADP- and thrombin-activated systems, respectively. With respect to the effects of clofazimine alone (in the absence of ADP or thrombin), slight augmentation of expression of CD62P was observed at 5 and 10 mg/L of this agent, the mean (±SD) values for the untreated control system and systems treated with 5 and 10 mg/L clofazimine being 1.09 ± 0.47%, 1.35 ± 0.63% and 1.48 ± 0.56%, respectively, (data from 6 experiments using PRP from 4 different donors).

Pre-treatment of PRP with α-tocopherol (30 mg/L) caused partial, albeit statistically significant, attenuation of clofazimine-mediated augmentation of ADP-activated expression of platelet CD62P, decreasing the mean (+ SD) levels of expression of CD62P from 44.3 ± 8.0 to 39.3 ± 7.2% (*P* < 0.05) and from 49.4 ± 8.1 to 42.2 ± 8.7% (*P* < 0.05) for systems treated with 0.625 and 1.25 mg/L clofazimine, respectively. The corresponding values for the clofazimine-free systems in the absence and presence of α-tocopherol were 35.0 ± 6.7% and 35.0 ± 7.1%, respectively, while the magnitude of CD62P expression on unstimulated platelets was 1.2 ± 0.4% (data from 8 separate experiments using PRP from 6 different donors).

With respect to isoniazid and rifampicin, the results for the unstimulated, and ADP-activated control system and systems treated with 5 and 10 mg/L of isoniazid are shown in Figure 1B, demonstrating lack of activity of these agents. Likewise, none of the test agents affected either spontaneous or thrombin-activated expression of CD62P (not shown).

### NP Aggregation

**FIGURE 2:**
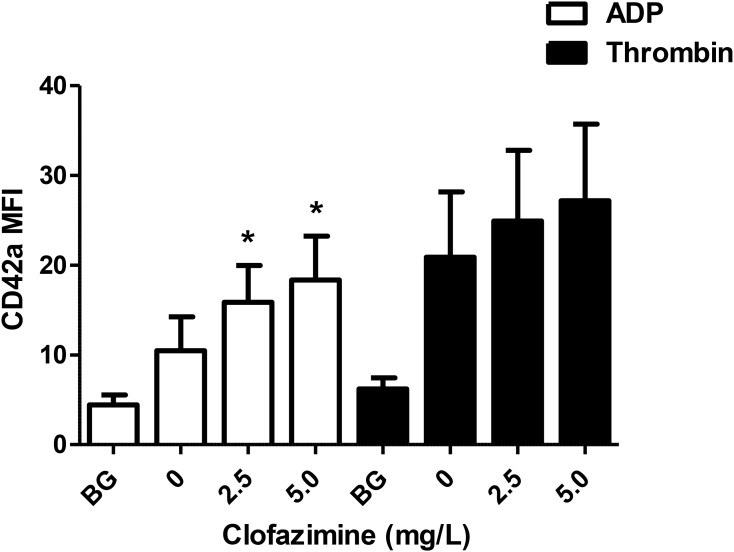
Effects of clofazimine at concentrations of 2.5 and 5 mg/L on the formation of neutrophil:platelet heterotypic aggregates following activation of buffy coat cell suspensions with ADP (100 μM; data from seven experiments using cells from seven different donors) or thrombin (0.31 NIH units/mL; data from 10 different experiments using cells from eight different donors). Results are expressed as the mean fluorescence intensity (MFI) of CD42a-PE emitted by CD16^+^/CD45^+^ neutrophils. ^∗^*P* < 0.05 for comparison of clofazimine-treated ADP- or thrombin-activated systems with the corresponding clofazimine-free control systems.

The effects of clofazimine on ADP- and thrombin-activated NP aggregation formation are shown in Figure 2. At both concentrations tested (2.5 and 5 mg/L), clofazimine caused significant potentiation of ADP-formation of NP aggregates. Although similar trends were observed with thrombin-activated systems, the differences between the control and clofazimine-treated systems did not achieve statistical significance.

## Discussion

The findings of the current study demonstrate that clofazimine, but not isoniazid or rifampicin, potentiates ADP- and thrombin-mediated activation of human platelets *in vitro*, measured according to the magnitude of upregulation of expression of the α-granule-derived adhesion molecule, CD62P, as well as ADP-activated formation of NP heterotypic aggregates. In this context, CD62P is widely recognized as being a key player in platelet-driven, systemic activation of neutrophils, as well as monocytes and vascular endothelium, effectively bridging thrombosis and inflammation (Wagner, 2005; Lisman, 2018). Importantly, the concentrations of clofazimine at which augmentation of platelet activation was observed are close to those reported to occur in serum following administration of this agent, ranging from 0.23 to 1.4 mg/L depending on dose and frequency of administration (Yawalkar and Vischer, 1979; Diacon et al., 2015). However, due to its lipophilicity (pKa = 8,51) and unusual pharmacokinetics, cell and tissue concentrations of clofazimine are considerably higher (Yawalkar and Vischer, 1979).

Although the mechanism of clofazimine-mediated augmentation of platelet activation remains to be established, it is nevertheless likely to be membrane-targeted and somewhat non-specific. This contention is based on the lipophilicity and cationic amphiphilic properties of clofazimine. These promote binding of cationic amphiphilic drugs to integral anionic membrane phospholipids, which anchor and maintain the functions of key membrane proteins in both eukaryotic and prokaryotic cells (Abraham et al., 2007; Nussio et al., 2007; Cholo et al., 2017). These events, in turn, lead to membrane destabilization, altered conformation of membrane proteins and loss of function, which, in the case of ion transporters, results in dissipation of the membrane potential and membrane depolarization. This scenario is supported by the observation that pre-treatment of platelets with α-tocopherol, an agent which attenuates the binding of cationic amphiphiles to phospholipids (Scuntaro et al., 1996), caused partial attenuation of clofazimine-mediated hyperreactivity of these cells. In this context, it is noteworthy that membrane depolarization has been reported to enhance receptor-mediated activation of platelets (Palés et al., 1988).

## Conclusion

Irrespective of putative mechanisms of action, the findings of the current study have identified a potentially adverse, pro-thrombotic activity of clofazimine, which, although unexplored in the clinical setting, may predispose patients with MDR/XDR-TB for development of cardiac dysfunction. This contention is of particular relevance in the context of possible synergistic cardiotoxicity resulting from multidrug therapy with clofazimine combined with agents such as bedaquiline and delaminid.This situation may be further exacerbated by the co-existent use of pro-atherosclerotic, anti-retroviral agents in settings with a high prevalence of HIV infection. Insights into mechanisms of these chemotherapy-related adverse events may provide a basis for future exploration of platelet-targeted therapies.

## Author Contributions

RA, AT, JN, CD, and GR were involved in performance of the various laboratory investigations. All authors contributed to the design of the study, analysis and interpretation of the data, and compilation of the manuscript.

## Conflict of Interest Statement

The authors declare that the research was conducted in the absence of any commercial or financial relationships that could be construed as a potential conflict of interest.
