# Supplementary material for: Clofazimine, but Not Isoniazid or Rifampicin, Augments Platelet Activation in vitro
Source: Front Pharmacol. 2018 Nov 20;9:1335. doi: 10.3389/fphar.2018.01335 (PMC6255828; doi:10.3389/fphar.2018.01335)
Supplement: Supplementary file 1 [file Table_1.DOCX]

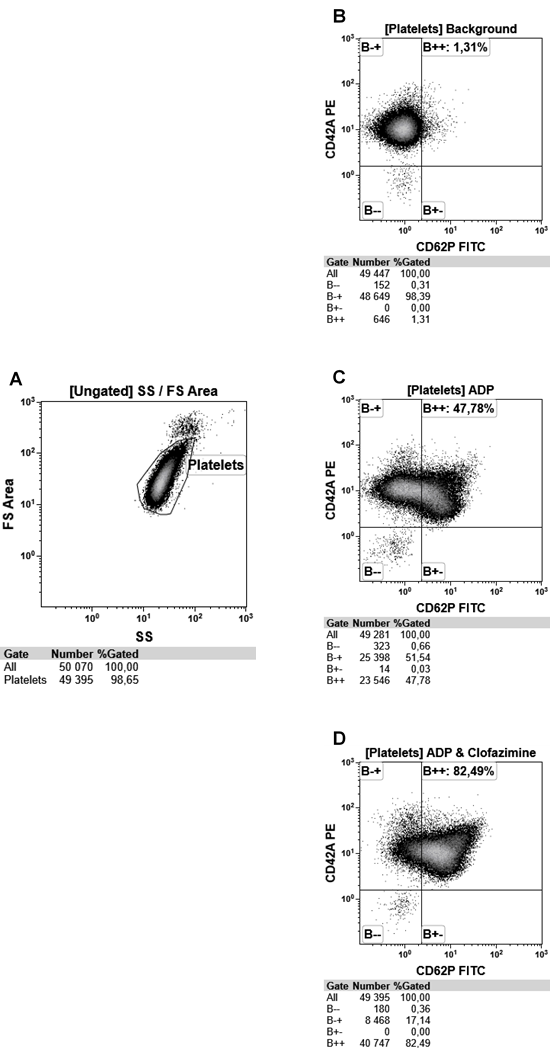


**Supplementary Figure 1: Representative flow cytometry density plots, indicating the gating and analysis strategy followed. A: A Forward Scatter (FS) Log vs Side Scatter (SS) Log density plot was used to identify the platelets. Discriminator (threshold) was set as channel 5, FS detector. Events outside region “platelets” were identified as residual CD45+ leukocytes. B-D: CD62P FITC vs CD42a PE Plots were used to determine the percentage activated platelets after gated on the “Platelet” region. All platelets were positive for CD42 PE, while activated platelets expressed CD62P PE. B: Represents data observed for untreated sample (background); C: Represents data observed for the ADP positive control; and D: Represents data observed for clofazimine (10 mg/L) in the presence of ADP.**
